# Severe Allergic Contact Dermatitis to Hair Dye in a Patient With Atopic Dermatitis During Dupilumab Therapy

**DOI:** 10.1111/cod.14759

**Published:** 2025-01-21

**Authors:** Alessandra Chiei Gallo, Gianluca Tavoletti, Gianluca Avallone, Eleonora Bono, Francesca Barei, Paolo Calzari, Angelo Valerio Marzano, Silvia Mariel Ferrucci

**Affiliations:** ^1^ Department of Pathophysiology and Transplantation University of Milan Milan Italy; ^2^ Dermatology Unit Fondazione IRCCS Ca' Granda Ospedale Maggiore Policlinico Milan Italy

**Keywords:** allergic contact dermatitis, atopic dermatitis, case report, dupilumab, hair dye, patch test

Dupilumab, a monoclonal antibody that targets the interleukin‐4 (IL‐4) receptor alpha, effectively disrupts the T helper 2 (Th2)‐mediated inflammatory cascade, a pathway critical to the pathogenesis of atopic dermatitis (AD). While its clinical benefits in AD are well‐documented, its influence on patch test outcomes and the management of allergic contact dermatitis (ACD) remains less clearly understood [1]. The immunological mechanisms driving ACD are complex, frequently involving a Th1/Th17‐mediated response [2]. In certain cases, specific allergens can activate different immune responses, such as the Th2 pathway triggered by exposure to fragrances and rubber, adding further complexity to the immunological landscape [3].

## 1 Case Report

A 38‐year‐old female with a longstanding history of AD presented with an Eczema Area and Severity Index (EASI) score of 26. Patient‐reported outcomes (PROs) included a Pruritus Numeric Rating Scale (Pruritus‐NRS) of 9, Sleep‐NRS of 9, Dermatology Life Quality Index (DLQI) of 21, and Hospital Anxiety and Depression Scale (HADS) scores of 9 for anxiety (HADS‐A) and 12 for depression (HADS‐D). The patient had been dyeing her hair regularly over the last year. Previous patch testing in 2018 (2016 SIDAPA Baseline series) was performed because of suspected contact triggers for her AD; paraphenylenediamine (PPD) 1% pet. was included but tested negative. Her medical history was significant for allergic asthma, rhinoconjunctivitis, bilateral keratoconus, and polysensitization to aeroallergens. The laboratory evaluation was largely unremarkable, with normal findings across complete blood count, inflammatory markers, and liver and kidney function tests, except for an elevated serum Immunoglobulin E (IgE) level of 2550 IU/mL. Previous treatments, including topical and systemic corticosteroids and calcineurin inhibitors, failed to achieve a sustained control. The patient initiated dupilumab with a 600 mg loading dose, followed by a 300 mg biweekly maintenance regimen, along with topical corticosteroids. At 16‐week, her EASI score had markedly improved to 2, Pruritus‐NRS to 5, with corresponding improvements in Sleep‐NRS (3), DLQI (2), HADS‐A (4), and HADS‐D (3).

**FIGURE 1:**
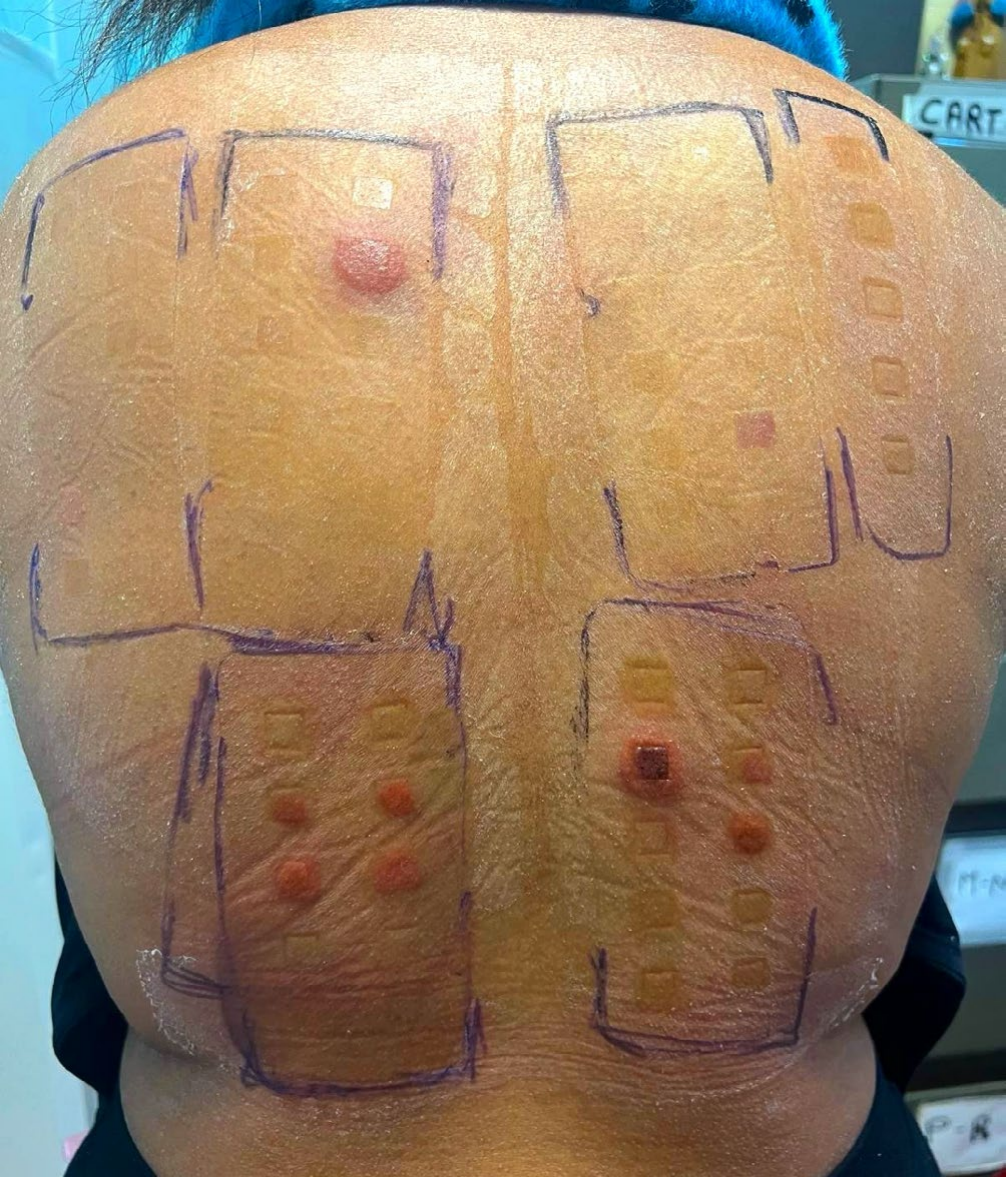
Positive patch test reactions (+++) characterised by marked erythema, induration, and papulation at multiple test sites, indicating strong sensitization to paraphenylenediamine 1% pet., p‐Aminophenol 1% pet., Disperse Orange 3 1% pet., Toluene‐2,5‐diamine‐sulphate 1% pet., and m‐Aminophenol 1% pet. The readings were performed on Day 2 and Day 4 after 48‐h occlusion, with reactions accompanied by bilateral axillary lymphadenopathy. The response persisted for 14 days and resolved completely with the daily application of topical methylprednisolone.

After six months of dupilumab, the patient experienced a worsening of AD symptoms a few days after exposure to hair dye (EASI 11, Sleep‐NRS 6, DLQI 5, HADS‐A 7, and HADS‐D 5), with eczematous lesions emerging on the neck, face and antecubital folds. Concurrently, erythematous and pruritic plaques developed on the scalp, raising suspicion of ACD potentially triggered by hair dye. Patch testing was recommended, employing the 2023 Italian Baseline series according to the AIFA Technical Committee [4], along with the hairdressing series (Smart Practice, Rome, Italy), performed without dupilumab discontinuation. Strongly positive reactions (+++) were observed to PPD 1% pet., p‐Aminophenol 1% pet., Disperse Orange 3 1% pet., Toluene‐2,5‐diamine‐sulphate 1% pet., and m‐Aminophenol 1% pet., applied for 48 h with day 2 and day 4 readings, (Figure 1), accompanied by bilateral axillary lymphadenopathy. Daily application of topical steroid therapy with methylprednisolone was initiated, resulting in resolution of clinical symptoms within 14 days. Strict allergen avoidance was initiated, leading to sustained remission of AD confirmed up to the 44‐week follow‐up.

## 2 Discussion

Emerging data offer varied insights into the impact of biologics on contact sensitization and patch testing outcomes [1]. Although dupilumab may improve skin barrier function [5] and theoretically reduce allergen penetration, recent reports show that ACD can still develop under dupilumab [6]. Of note, although newly positive reactions raise the question of primary sensitization during dupilumab therapy, we cannot exclude that sensitization may have occurred before initiation of dupilumab or due to repeated hair dye exposures. This case thus illustrates that dupilumab neither necessarily prevents the onset of ACD nor suppresses strong patch test reactions in sensitised individuals. These observations are in line with recent literature highlighting that patch testing remains a reliable diagnostic tool during dupilumab therapy and can reveal strong reactions [7, 8, 9].

In conclusion, clinicians should remain vigilant for contact sensitization in patients undergoing dupilumab therapy, especially when eczema flare‐ups or incomplete responses are observed. Ongoing research is needed to clarify the interplay between dupilumab and ACD, as well as to optimise management strategies for patients with coexisting AD and ACD.

## Author Contributions

**Alessandra Chiei‐Gallo:** writing – original draft, conceptualization, writing – review and editing. **Gianluca Tavoletti:** writing – review and editing, writing – original draft, conceptualization. **Gianluca Avallone:** writing – review and editing, methodology. **Eleonora Bono:** data curation, conceptualization. **Francesca Barei:** writing – review and editing. **Paolo Calzari:** writing – review and editing. **Angelo Valerio Marzano:** supervision, writing – review and editing. **Silvia Mariel Ferrucci:** supervision, conceptualization, methodology, writing – review and editing.

## Ethics Statement

The patient described in this paper has given her written informed consent to the publication of the case details.

## Conflicts of Interest

The authors declare no conflicts of interest.

## Data Availability

Data supporting the findings of this study are available on request from the corresponding author.
